# Effects of Prepartum Immunotropic Treatment on Growth Performance, Physiological Status, and Early-Life Adaptation of Holstein Calves

**DOI:** 10.3390/ani16121916

**Published:** 2026-06-20

**Authors:** Ainur Davletova, Malika Shamekova, Vladimir Semenov, Andrey Klyapnev, Serimbek Abugaliev, Adilbek Zholdasbekov, Darkhan Smagulov, Yedige Nassambayev, Maxat Toishimanov, Dastanbek Baimukanov

**Affiliations:** 1 West Kazakhstan Innovation and Technological University, Uralsk 090000, Kazakhstan; 2 Laboratory of Breeding and Biotechnology, Institute of Plant Biology and Biotechnology, Timiryazev 45, Almaty 050040, Kazakhstan; 3 Department of Morphology, Obstetrics and Therapy, Chuvash State Agrarian University, K. Marx str., 29, Chuvash Republic, Cheboksary 428003, Russia; 4 Department of Anatomy, Surgery and Internal Noncommunicable Diseases, Nizhny Novgorod State Agricultural Academy, 97 Prospect Gagarina, Nizhny Novgorod 603107, Russia

**Keywords:** Holstein calves, immunotropic preparations, Ribotan, sodium nucleinate, growth performance, hematology, biochemical parameters, cortisol, early-life adaptation, dairy cattle

## Abstract

The present study evaluated the effects of the prepartum administration of immunotropic preparations on the growth performance, physiological status, and metabolic profile of calves. Sixty pregnant Holstein cows were divided into three groups (*n* = 20 each): the first experimental group received a single intramuscular injection of sodium nucleinate (5 mL), the second experimental group received a single intramuscular injection of Ribotan (5 mL), and the control group received saline solution. All treatments were administered 3–9 days before calving. The obtained calves were monitored until 60 days of age. Clinical, growth, hematological, and biochemical parameters were assessed at days 1, 10, 30, and 60. Calves from the treated cows showed improved neonatal adaptation, including faster development of standing posture and the suckling reflex. Body weight was significantly higher in experimental groups at 30 and 60 days (*p* ≤ 0.05), with consistently greater average daily gains. Blood analysis revealed increased total protein, albumin, and γ-globulin levels, indicating enhanced protein metabolism and immune status. In contrast, cortisol concentrations were lower in treated groups, reflecting reduced physiological stress. Multivariate (PCA) and correlation analyses confirmed strong associations between growth performance, metabolic activity, and immune indicators, and demonstrated clear separation between control and treated groups. Ribotan exhibited the most pronounced biological effect, while sodium nucleinate showed moderate but consistent improvements. In conclusion, prepartum immunotropic treatment of cows enhances early-life adaptation, metabolic efficiency, and growth performance of calves and may represent a practical strategy for improving calf rearing outcomes in dairy farming systems.

## Simple Summary

Improving calf health and growth during early life is essential for dairy production efficiency. This study investigated whether immunotropic preparations administered to cows before calving could enhance the physiological status and growth of their offspring. The results demonstrated that such treatments, particularly Ribotan, improved adaptation, reduced stress, and promoted better growth in calves.

## 1. Introduction

Dairy cattle breeding in Kazakhstan is a dynamically developing sector of productive animal husbandry. The improvement of productive traits is achieved through the use of semen from Holstein cattle bulls of foreign selection [1,2,3].

Animal organisms are influenced by climatic factors, including temperature, humidity, and seasonal and weather-related variations, as well as environmental factors such as housing conditions, air quality, and other management-related parameters, which may adversely affect animal health [4,5].

The neonatal period—defined as the first month of life—is the most hazardous stage in a calf’s development, owing to the immaturity of the neonatal immune system and the high environmental burden of infectious agents [6]. Gastrointestinal and respiratory infections are the leading causes of morbidity and mortality in newborn calves, followed by abomasal, umbilical, and developmental disorders, all of which compromise subsequent growth and lifetime productivity. In dairy production, neonatal losses not only increase rearing costs but also reduce future reproductive performance and the first-lactation milk yield of heifers, making early-life health a decisive factor in herd profitability [7].

Because epitheliochorial placentation in cattle prevents prenatal transfer of maternal immunoglobulins, the newborn calf is born essentially agammaglobulinemic and depends entirely on the timely ingestion and absorption of colostral antibodies to acquire passive immunity. Failure of passive transfer (FPT), commonly defined as serum IgG below 10 g/L at 24–48 h after birth, is strongly associated with elevated rates of diarrhea, pneumonia, septicemia, and death during the first weeks of life [8,9,10]. Consequently, strategies that enhance the quality of colostrum and strengthen the immunological competence of the dam during the dry and prepartum periods are of major applied importance for modern dairy farming [11,12].

Immediately after birth, the organism of calves is exposed to the external environment for the first time. This is accompanied by the morphofunctional restructuring of the cardiovascular, respiratory, digestive, hematopoietic, immune, and urinary systems.

Nutrition plays a crucial role in the life of newborn calves. Its quality determines all biochemical processes in the body as well as growth performance. During the first week of life, sawtooth-like growth curves of body weight are observed due to daily fluctuations, with peaks occurring after feeding and minima before the subsequent feeding [13].

Colostrum is the primary source of nutrition for newborn calves. Studies comparing calves fed fresh or frozen colostrum, or deprived of colostrum, showed that γδ T cells increased during the first two days in all groups; however, their maintenance was impaired in colostrum-deprived calves, accompanied by a compensatory rise in CD4+ and CD8+ T cells. Neutrophils in these calves exhibited a more pro-inflammatory profile. Colostrum deprivation also altered T- and B-cell as well as monocyte profiles and affected immune memory development. Although both fresh and frozen colostrum supported IgG transfer, frozen colostrum modified the maturation of γδ memory T cells, suggesting that processing methods influence immune development [14].

Calves in the early postnatal period are highly susceptible to diarrhea. Studies in calves up to 13 days of age showed that, in the three days preceding disease onset, serum IgG, triglycerides, insulin, cholesterol, and plasma osmolality were reduced, while fecal pH increased. After the onset of diarrhea, fecal short-chain fatty acid concentrations decreased, plasma glucose levels declined, and plasma urea levels increased. No significant changes were observed in non-esterified fatty acids, total protein, acute-phase proteins, or liver enzyme activity. These early alterations in blood and fecal parameters prior to the appearance of clinical signs suggest adaptive responses to subclinical digestive stress. Therefore, optimization of feeding and management strategies is essential to mitigate gastrointestinal disorders in newborn calves [15,16,17,18].

Among such strategies, the prepartum immunomodulation of pregnant cows has attracted growing interest. Specific immunization of cows in late gestation significantly increases IgA, IgG, and pathogen-specific antibody concentrations in their offspring, confirming that the immune status of the dam directly shapes neonatal humoral protection [19]. Non-specific immunomodulating feed additives administered during the dry period have likewise been shown to improve innate immune indices, increase birth weight, and support the postnatal growth of calves, particularly under heat-stress conditions [12]. Probiotic and microbial-based immunostimulants have produced analogous effects, attenuating respiratory inflammation and enhancing mucosal IgA responses in calves [20].

Two preparations—sodium nucleinate and Ribotan—are widely used as non-specific immunomodulators in cattle. Sodium nucleinate, a low-molecular-weight nucleic-acid derivative, induces leukocyte reactions, stimulates intracellular and nucleic-acid metabolism, exerts anti-inflammatory activity, and is particularly effective in correcting immunodeficiency states in animals [21]. Ribotan, a complex preparation of low-molecular-weight polypeptides (0.5–1 kDa) and RNA fragments, stimulates both T- and B-cell immunity, enhances macrophage activity and interferon/lymphokine synthesis, reduces stress reactions, and is recommended for the prevention and therapy of viral, bacterial, and parasitic diseases in cattle [22,23]. Despite their broad clinical application, comparative data on the effects of these two immunotropic agents when administered to cows in the immediate prepartum period on the growth, hematological, biochemical, and adaptive parameters of their offspring remain limited.

Given the documented links between maternal immune status, colostrum quality, passive transfer, and calf viability, elucidating how prepartum administration of sodium nucleinate versus Ribotan influences early-life adaptation of Holstein calves is both scientifically relevant and practically important. Therefore, the aim of the present study was to evaluate the effects of a single prepartum intramuscular injection of sodium nucleinate or Ribotan to Holstein cows 3–9 days before calving on the growth performance, clinical and physiological status, and hematological and biochemical indices of their calves during the first 90 days of life, under the zoohygienic conditions of a commercial dairy farm in the West Kazakhstan region.

## 2. Materials and Methods

### 2.1. Study Site and Ethical Approval

The study was conducted from November 2024 to March 2025 at the Arystanov dairy farm located in the Baiterek district of the West Kazakhstan region. All animals were maintained under identical housing, feeding, and management conditions throughout the study period.

All experimental procedures involving animals were approved by the Coordination Council of the West Kazakhstan Innovation and Technological University (Protocol No. 3–2024).

### 2.2. Experimental Design and Animals

The experimental design was based on the analog pair method [24]. Sixty pregnant Holstein cows were selected and divided into three groups (*n* = 20 per group), balanced by age, milk productivity, and body weight: two experimental groups and one control group.

Cows in the first experimental group received a single intramuscular injection of 5 mL of a 0.2% aqueous solution of sodium nucleinate. Cows in the second experimental group were administered a single intramuscular injection of 5 mL of Ribotan solution. Control animals received 5 mL of 0.9% sodium chloride solution. All treatments were applied 3–9 days before calving.

Calves obtained from these cows were assigned to the same groups as their dams. In the offspring, clinical, physiological, growth, hematological, and biochemical parameters were evaluated at days 1, 10, 30, and 60 of life.

### 2.3. Feeding and Management

All cows were maintained under identical housing and feeding conditions throughout the study, as shown in Table 1. During the dry period, cows received a daily ration consisting of 18 kg silage, 3 kg straw, 2.5 kg barley grain, and 0.05 kg premix. Following calving, lactating cows were fed 23 kg silage, 4 kg alfalfa hay, 5 kg barley grain, 2.5 kg maize grain, 2.5 kg rapeseed meal, 0.15 kg premix, and 0.2 kg tricalcium phosphate per animal per day. Fresh drinking water was available ad libitum. Calves were fed whole milk according to the farm management protocol. The feeding regimen was identical for all experimental groups to eliminate nutritional effects on calf growth and physiological responses.

### 2.4. Zootechnical and Clinical Measurements

Body weight of calves was measured monthly, and average daily gain (ADG) was calculated. Clinical and physiological parameters, including rectal temperature, heart rate, and respiratory rate, were determined according to standard physiological methods.

### 2.5. Hematological Analysis

Hematological parameters, including hemoglobin concentration, hematocrit, erythrocyte, leukocyte, and platelet counts, were determined using an automated hematology analyzer (HTI Micro-CC-20 Plus, High Technology Inc., North Attleboro, MA, USA). Blood smears were stained according to the Romanowsky method, and leukocyte differential counts were performed.

### 2.6. Biochemical Analysis

Biochemical analyses were performed using standard laboratory methods. Total serum protein was measured using an ICUBIO iMagic-V7 analyzer (Shenzhen iCubio Biomedical Technology Co., Ltd., Shenzhen, China), and protein fractions (albumin, α-, β-, and γ-globulins) were determined using a Minicap analyzer (Sebia, Lisses, France). Glucose concentration was determined by the orthotoluidine method, urea by the diacetyl monoxime method, total calcium by complexometric titration, and inorganic phosphorus using a vanadate–molybdate reagent. Cortisol levels were measured using a commercial ELISA kit (“Cortisol-IFA K210”). Blood analyses were conducted at the Agrarian Innovation and Technology Park of the West Kazakhstan Innovation and Technological University.

### 2.7. Statistical Analysis

All data were processed using standard statistical methods. Descriptive statistics are presented as mean ± standard error of the mean (M ± m). To evaluate the effects of treatment (control, Ribotan, sodium nucleinate) and time (days 1, 10, 30, and 60). The effects of treatment group, sampling day, and their interaction were evaluated using two-way ANOVA.

Differences were considered statistically significant at *p* ≤ 0.05. In tables, different superscript letters (a–c) within the same row indicate significant differences between groups at a given time point. Multivariate analysis was conducted using principal component analysis (PCA) to identify patterns and relationships among growth, hematological, biochemical, and hormonal parameters. Correlation analysis was performed using Pearson’s correlation coefficient to assess associations between variables, and results were visualized as a heatmap.

All statistical analyses were performed using JMP Pro 17.

## 3. Results

Clinical examination revealed that the administration of immunotropic preparations to pregnant cows had a positive effect on the physiological status of newborn calves. On day 2 of life, rectal temperature in the control group was 38.5 ± 0.15 °C, whereas calves from the experimental groups exhibited significantly higher values (39.1 ± 0.12 and 39.6 ± 0.16 °C), which may reflect more intensive metabolic and oxidative processes. However, at days 10 and 30, body temperature in the experimental groups was lower by 0.2–0.6 °C compared to the control, likely due to a reduced incidence of diseases such as diarrhea and bronchopneumonia in treated animals.

According to periodic weighing data, body weight increased progressively with age in all groups (Table 2). Two-way ANOVA demonstrated significant effects of treatment group (*p* < 0.0001), sampling day (*p* < 0.0001), and treatment × day interaction (*p* < 0.0001), indicating that growth responses differed among groups over time. At birth, body weight did not differ significantly among groups (*p* > 0.05). However, from day 30 onward, calves from both experimental groups exhibited significantly greater body weight than control animals (*p* < 0.05). By day 60, body weight reached 74.83 ± 0.50 kg and 74.50 ± 0.46 kg in the Ribotan and sodium nucleinate groups, respectively, compared with 72.40 ± 0.36 kg in the control group. Ribotan consistently produced the highest body weight values throughout the study. Average daily gain followed a similar pattern. Significant effects of treatment group (*p* = 0.0008) and sampling day (*p* < 0.0001) were observed, whereas the interaction effect was not significant. At day 30, ADG was significantly higher in calves from the Ribotan (636 g) and sodium nucleinate (628 g) groups than in controls (592 g), and this advantage persisted through day 60, confirming a sustained stimulatory effect of both immunotropic preparations on growth performance.

Erythrocyte count (RBC) was significantly influenced by treatment group (*p* = 0.0003) and sampling day (*p* < 0.0001), whereas the interaction effect was not significant. At day 1, RBC values were higher in the control group than in calves from the sodium nucleinate group (*p* < 0.05), while the Ribotan group exhibited intermediate values. Subsequently, erythrocyte counts gradually converged, and by day 60 only minor differences were observed among groups. In contrast, hemoglobin concentration was significantly affected by sampling day (*p* < 0.0001), whereas treatment group and treatment × day interaction had no significant effects. Hemoglobin values declined during the first month of life and subsequently increased toward day 60 in all groups, reflecting normal physiological adaptation during calf development (Table 3).

Leukocyte count (WBC) was strongly affected by treatment group, sampling day, and their interaction (all *p* < 0.0001). Calves from the Ribotan group exhibited the highest WBC concentrations throughout the study, particularly during the first 10 days of life, indicating enhanced activation of immune responses. Although WBC values decreased with age in all groups, treated calves maintained significantly higher leukocyte counts than controls at most observation points, suggesting a prolonged immunostimulatory effect of maternal treatment.

Total protein concentration was significantly influenced by treatment group, sampling day, and treatment × day interaction (all *p* < 0.0001), as shown in Table 4. At all sampling times, calves from treated cows exhibited higher total protein concentrations than controls, with the highest values observed in the Ribotan group. Albumin concentration was similarly affected by treatment group (*p* < 0.0001) and sampling day (*p* < 0.0001), whereas the interaction effect was not significant. The consistently greater albumin concentrations observed in treated calves indicate enhanced protein metabolism and improved nutritional status.

Pronounced differences were observed in serum globulin fractions. α-Globulin concentrations were significantly affected by treatment group (*p* < 0.0001) and sampling day (*p* < 0.0001), with values decreasing progressively over time in all groups. β-Globulins were significantly influenced by treatment group (*p* = 0.0004), sampling day (*p* < 0.0001), and treatment × day interaction (*p* = 0.0187). The highest β-globulin concentrations were generally recorded in calves from the Ribotan group during the early postnatal period. The most pronounced treatment effect was observed for γ-globulins, for which significant effects of treatment group, sampling day, and interaction were detected (all *p* < 0.0001). Calves from both experimental groups exhibited substantially higher γ-globulin concentrations than controls from the first day of life onward, indicating enhanced humoral immune status. This effect remained evident throughout the study and was most pronounced in the Ribotan-treated group.

Glucose concentration was significantly affected by treatment group (*p* < 0.0001) and sampling day (*p* < 0.0001), whereas the interaction effect approached significance (*p* = 0.0536). Glucose levels decreased with age in all groups; however, treated calves consistently maintained higher concentrations than controls, particularly during the later stages of the experiment. Urea concentration was significantly influenced by sampling day (*p* < 0.0001) and treatment × day interaction (*p* = 0.0123), whereas the main effect of treatment group was not significant. These results indicate that age-related changes in protein metabolism differed among treatments despite similar overall urea concentrations.

Mineral metabolism was also affected by maternal immunotropic treatment. Calcium concentration was significantly influenced by treatment group (*p* < 0.0001) and treatment × day interaction (*p* = 0.0040), whereas the effect of sampling day was not significant. Treated calves generally exhibited higher calcium concentrations than controls, with the highest values observed in the Ribotan group. Phosphorus concentration was significantly affected by treatment group (*p* = 0.0002), whereas neither sampling day nor the interaction effect reached statistical significance, indicating a stable treatment-related difference throughout the observation period.

Cortisol concentration demonstrated marked differences among groups and across time. Significant effects of treatment group, sampling day, and treatment × day interaction were detected (all *p* < 0.0001). Cortisol concentrations were highest during the first day of life and declined substantially thereafter in all groups. Although calves from treated cows initially exhibited elevated cortisol concentrations, a more rapid decline was observed during the subsequent observation periods. By day 60, cortisol concentrations in both treated groups were lower than in the control group, indicating improved physiological adaptation and reduced stress responses. The lowest cortisol concentrations were consistently observed in calves originating from Ribotan-treated cows.

**Figure 1:**
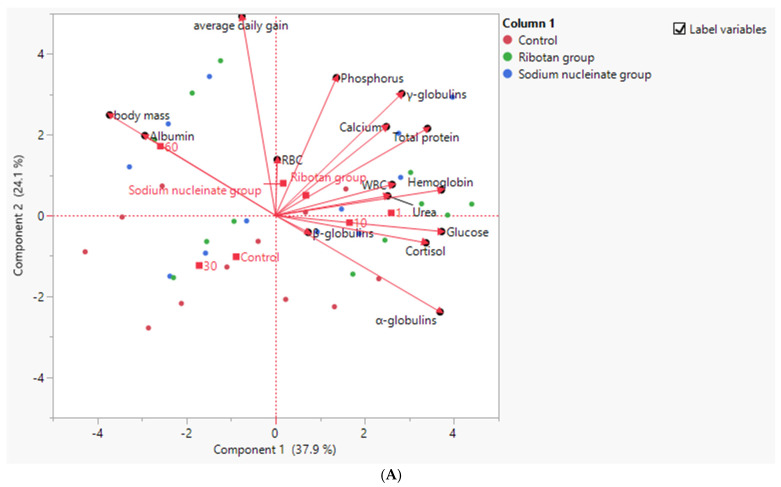

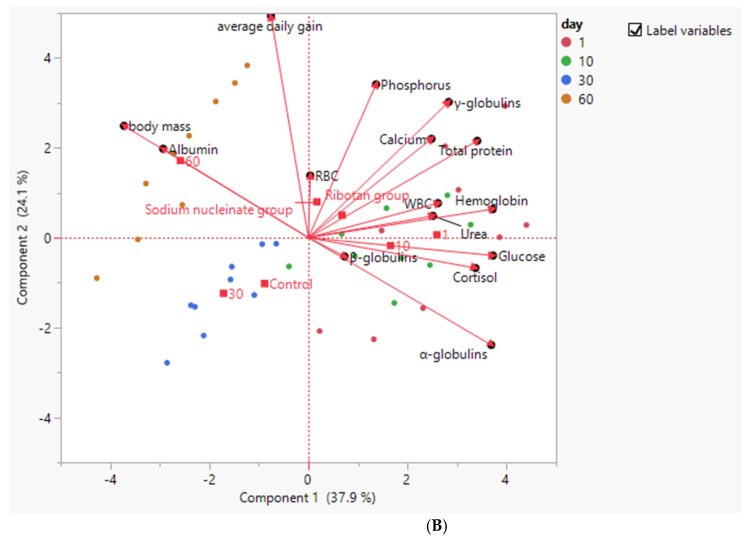
Principal component analysis (PCA) score and loading plots showing the distribution of samples by (**A**) treatment group and (**B**) experimental day.

PCA revealed a clear separation of samples according to both treatment and time, with the first two principal components explaining a substantial proportion of the total variance (PC1: 37.9% and PC2: 24.1%). Along PC1, the control group was primarily distributed on the negative axis, whereas calves treated with Ribotan and sodium nucleinate were shifted toward positive values, indicating an improved physiological and metabolic status under immunomodulator administration, with Ribotan showing the most pronounced effect. This separation was mainly driven by variables with strong positive loadings, including glucose, WBC, total protein, γ-globulins, and hemoglobin, all of which are associated with enhanced metabolic activity and immune function, while the control group was characterized by comparatively lower values of these indicators. Temporal dynamics were also evident, as early time points (days 1 and 10) clustered closer together, reflecting similar initial physiological conditions, whereas later stages (days 30 and 60) were clearly separated, indicating progressive metabolic differentiation over time (Figure 1B). Day 60 samples were strongly associated with increased body mass, albumin, and phosphorus, reflecting advanced growth and improved metabolic status, while early stages were linked to higher cortisol levels, suggesting stress adaptation after birth. The loading plot further demonstrated that γ-globulins, total protein, and hemoglobin contributed strongly to positive PC2 values, whereas glucose and WBC were dominant contributors to PC1, and β-globulins and cortisol were positioned in the opposite quadrant, indicating inverse relationships with metabolic improvement. Additionally, the alignment of body mass and albumin vectors confirmed a strong positive correlation between growth performance and protein metabolism. Overall, PCA supported the univariate statistical results and demonstrated that immunomodulator treatments, particularly Ribotan, induced consistent and biologically meaningful improvements in growth, metabolic activity, and immune status, with these differences becoming more pronounced over time (Figure 1A).

PCA results were consistent with the univariate ANOVA and Tukey’s HSD test, confirming the robustness of the observed differences between groups. The clear separation of the control and treated groups along PC1 (37.9%) reflects the statistically significant differences identified by ANOVA in key variables such as body mass, average daily gain, total protein, albumin, WBC, and γ-globulins (*p* < 0.05), which also showed distinct Tukey groupings (a–c). In particular, variables with high positive loadings on PC1 (glucose, WBC, total protein, γ-globulins, and hemoglobin) correspond to those parameters where the Ribotan and sodium nucleinate groups significantly exceeded the control according to Tukey’s test, thereby driving the multivariate separation. The intermediate position of the sodium nucleinate group in the PCA plot is also in agreement with Tukey results, where this group often shared letters with both the control and Ribotan groups, indicating partial but not always complete statistical differentiation. Furthermore, the temporal gradient observed in PCA, with later time points (days 30 and 60) clearly separated from early stages (days 1 and 10), corresponds to the increasing number of statistically significant differences over time detected by ANOVA, particularly for growth performance and protein metabolism indicators. Variables located in opposing quadrants of the loading plot, such as cortisol and β-globulins, showed inverse relationships with the main metabolic axis and were also characterized by significant differences in ANOVA at specific time points, supporting their contribution to group discrimination. Thus, the PCA not only visualizes the multivariate structure of the dataset but also integrates and confirms the statistical significance patterns identified by ANOVA and Tukey’s HSD test, demonstrating that the observed treatment effects are systematic, biologically meaningful, and consistent across analytical approaches.

Correlation analysis revealed strong relationships between growth performance, hematological, and biochemical parameters, confirming the integrated nature of metabolic and physiological processes in calves. Body mass showed a very strong positive correlation with average daily gain (r = 0.9536), indicating consistency between cumulative and incremental growth indicators. In addition, body mass was strongly positively associated with albumin (r = 0.7692), suggesting a close link between growth intensity and protein metabolism. Conversely, body mass demonstrated strong negative correlations with α-globulins (r = −0.9276), glucose (r = −0.7320), and cortisol (r = −0.6619), indicating that increased growth is associated with reduced stress levels and a shift in protein fractions toward anabolic processes.

Average daily gain was strongly positively correlated with γ-globulins (r = 0.9610) and RBC (r = 0.7416), reflecting the importance of immune status and oxygen transport in growth processes. A strong negative relationship between average daily gain and α-globulins (r = −0.9058) further supports the transition from inflammatory or stress-related responses toward productive metabolism in rapidly growing animals.

**Figure 2:**
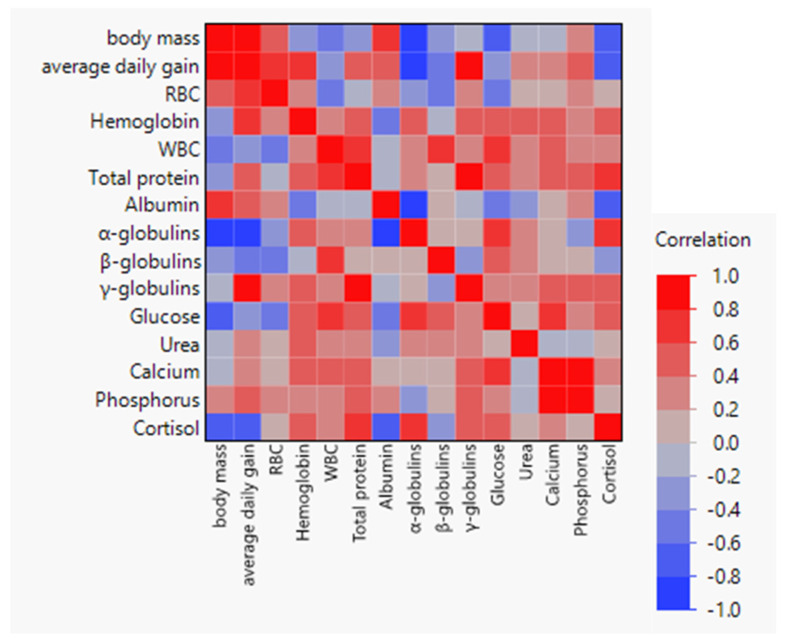
Correlation heatmap of growth performance, hematological, biochemical, and hormonal parameters in calves across the experimental groups.

Total protein showed strong positive correlations with γ-globulins (r = 0.8531), WBC (r = 0.6830), and glucose (r = 0.5771), indicating that protein metabolism is closely linked to immune activity and energy supply. Hemoglobin was moderately positively correlated with glucose (r = 0.5601), calcium (r = 0.5301), and cortisol (r = 0.5206), suggesting interactions between oxygen transport, mineral metabolism, and endocrine regulation (Figure 2).

Glucose demonstrated strong positive correlations with calcium (r = 0.6657) and WBC (r = 0.6804), reflecting the coupling between energy metabolism, mineral balance, and immune activation. Calcium and phosphorus were highly correlated (r = 0.8706), indicating coordinated regulation of mineral metabolism and skeletal development.

Cortisol showed strong positive correlations with α-globulins (r = 0.7635) and total protein (r = 0.6687), and negative correlations with body mass (r = −0.6619) and albumin (r = −0.6203), confirming its role as a stress marker negatively associated with growth and anabolic processes.

## 4. Discussion

The present study demonstrated that prepartum administration of immunotropic preparations—sodium nucleinate and Ribotan—to pregnant Holstein cows produced measurable and biologically consistent improvements in the clinical, physiological, hematological, and biochemical status of their offspring, as well as in growth performance during the first 60 days of life. The present study demonstrates that a single prepartum intramuscular injection of sodium nucleinate or Ribotan to Holstein cows 3–9 days before calving exerts a pronounced and sustained positive effect on the growth performance, clinical status, and hematological and biochemical profile of their calves during the first 60 days of life. These findings are consistent with the concept that maternal immune competence in late gestation is a key determinant of colostrum quality, passive immunity, and subsequent neonatal adaptation [25,26,27].

A central observation of our work is the markedly higher γ-globulin and total protein concentrations in calves from both experimental groups from the very first day of life. Because γ-globulin level in neonatal serum is a direct biochemical proxy for IgG and reflects successful passive transfer of colostral antibodies [28,29], the elevated γ-globulin values in the Ribotan and sodium nucleinate groups indicate enhanced colostral immunoglobulin transfer, most likely due to improved immunological competence of the dam in the days preceding parturition. Similar stimulation of specific and non-specific antibody production in calves born to immunologically primed or immunomodulated cows has previously been reported for vaccinated pregnant cows [16] and for cows supplemented with OmniGen-AF during the dry period [12]. Given that serum IgG < 10 g/L is a well-established threshold for failure of passive transfer and is associated with higher rates of diarrhea and pneumonia [10], the sustained elevation of γ-globulins in treated calves likely explains their improved clinical status.

The clinical and behavioral indicators of early adaptation—shorter latency to stable standing, earlier onset of the suckling reflex, and more physiological heart and respiratory rates—further support this interpretation. Faster achievement of the suckling reflex is directly linked to earlier and greater colostrum intake, which in turn improves passive immune transfer and reduces morbidity [30]. The transiently higher rectal temperature on day 2 in the treated groups may reflect more intense metabolic and oxidative activity associated with accelerated postnatal adaptation, while the lower temperatures and heart rates from day 10 onward are consistent with a lower incidence of subclinical inflammation and diarrheal/respiratory disease in these animals [31,32].

Growth performance was significantly enhanced in both treated groups, with Ribotan consistently producing the largest effect. By day 60, calves from the Ribotan and sodium nucleinate groups were 3.46 and 2.87 kg heavier than controls, respectively, and exhibited significantly higher ADG from day 30 onward. These results are in line with previous reports showing that strategies which improve passive immunity and immune function (colostrum doubling, probiotic/prebiotic supplementation, immunomodulating feed additives) lead to increased ADG in preruminant and weaned calves [33,34].

A higher serum total protein in early life is also a recognized predictor of superior growth rate in dairy calves, which is fully consistent with the parallel increases in total protein, albumin, and body mass observed in our experimental groups. In a large-scale study involving 39,619 neonatal Holstein, Jersey, and crossbred calves, Cortese et al. [35] demonstrated that a serum total protein concentration between 6.0 and 8.5 g/dL was optimal for health and growth, and that average daily gain over the first 120 days of life increased significantly as serum total protein concentration rose within this range. Similarly, Aghakhani et al. [36] showed in 152 female Holstein calves that those with total serum protein above 6.5 g/dL at 24 h of age had significantly greater body weight at days 30 and 60 and higher average daily gains during the first two months of life compared to calves with lower protein concentrations. The total protein values achieved in our Ribotan and sodium nucleinate groups (65–73 g/L throughout the study) fall squarely within the range associated with superior growth in these investigations, lending strong external validity to our growth performance findings.

The hematological response showed characteristic patterns of immunostimulation. Leukocyte counts were significantly elevated in the Ribotan group at day 1 and day 10, indicating rapid mobilization of cellular defense mechanisms, similar to leukocytosis observed in cattle following administration of other immunostimulants [37]. This finding is consistent with the results of Dudek et al. [38], who reported that pegbovigrastim administration to healthy 5-week-old calves significantly increased the total leukocyte count and the counts of all examined leukocyte subsets within days of injection, accompanied by enhanced phagocytic activity; and with Mukhutdinova et al. [10], who similarly documented significant increases in leukocyte indices in calves born to cows treated prepartum with Ribotan and other immunostimulants in combination with a mineral additive. The transient reduction in erythrocyte count and hemoglobin at day 30 in treated animals, followed by full recovery and even superior hemoglobin values at day 96, likely reflects physiological hemodilution during intensive growth rather than anemia, as calcium, phosphorus, and total protein levels remained simultaneously high [39,40,41]. This pattern mirrors the age-related erythrocyte and hemoglobin dynamics documented by Mohri et al. [37] in Holstein calves during the first months of life, in which transient physiological decreases in red blood cell parameters were associated with accelerated growth rather than pathological anemia. The sustained elevation of β-globulins in early life, together with high γ-globulins, supports activation of transport and humoral immune proteins by the tested preparations, in agreement with the known mechanisms of action of ribonucleic-acid- and polypeptide-based immunomodulators [42,43]. Dinardo et al. [18] likewise reported that oral nucleotide supplementation in Holstein Friesian calves stimulated immunoglobulin-related serum protein fractions and supported immune development during the first 25 days of life, underscoring the capacity of nucleic-acid-based preparations to modulate the humoral immune compartment in early life.

Biochemical shifts also indicate improved metabolic and mineral homeostasis. Significantly higher glucose concentrations in treated calves from day 30 onward suggest enhanced energy supply for growth, whereas the slightly lower urea values may reflect more efficient utilization of amino acids for anabolic processes rather than their deamination. These findings are in agreement with observations by Ockenden et al. [44], who demonstrated in Holstein heifer calves that accelerated preweaning nutrition was associated with significantly higher blood glucose and insulin concentrations at the time of an immune challenge, alongside superior growth rates and leukocyte counts, indicating a functional coupling between energy metabolism and immune activation that parallels the relationships observed in the present study. The higher calcium and phosphorus concentrations in Ribotan- and sodium nucleinate-treated calves, particularly at day 60, are consistent with improved mineral metabolism and skeletal development and mirror the body-weight advantage of these groups. Similar coupling between protein metabolism, mineral status, and growth has been described in calves with robust passive immunity and favorable early-life health [45,46].

Cortisol dynamics provide additional mechanistic insight. The significantly higher cortisol concentrations in treated calves on day 1 most likely represent an adaptive “eustress” response typical of the immediate postnatal period, when elevated cortisol promotes maturation of endocrine and metabolic systems and supports transition to extra-uterine life [47]. The transient postnatal cortisol peak observed in our treated groups is consistent with the findings of Kirovska et al. [47], who documented a physiological surge in cortisol in healthy newborn calves immediately after birth, and with Arfuso et al. [48], who reported a strong positive correlation (r  =  0.83) between periparturient cortisol concentrations in Simmental cows and those in their calves at day 1 of life, confirming that the initial postnatal hormonal response is tightly linked to the maternal endocrine environment. The subsequent sharp decline and consistently lower cortisol in experimental groups from day 10 onward indicate a more rapid attenuation of physiological stress and improved adaptive capacity, in agreement with the established role of cortisol as a negative modulator of innate immunity and growth in peripartum cows and neonatal calves [48]. The strong negative correlation observed in our data between cortisol and body mass or albumin, and the positive correlation between cortisol and α-globulins, support this interpretation and indicate a shift from stress-related catabolic responses toward anabolic, growth-oriented metabolism in treated animals. This is consistent with observations by Masmeijer et al. [49], who studied veal calves at arrival and found that above-average cortisol concentrations at arrival were associated with below-average body weight, failure of passive immune transfer, and an acute-phase response, while calves with lower cortisol levels formed a low-risk cluster with superior body weight and immune status—an inverse relationship that closely mirrors the pattern we observed between day 10 and day 60 in treated versus control calves.

Comparison of the two preparations indicates that Ribotan, a complex low-molecular-weight polypeptide–RNA immunomodulator acting on both innate and adaptive immunity and on interferon/lymphokine synthesis [19,50], produced the strongest and most consistent effects across growth, protein, mineral, and stress indicators. This superiority is consistent with a study by Mukhutdinova et al. [10], who compared Ribotan, Immunoferon, and therapeutic-prophylactic immunoglobulin in combination with the mineral additive Felutsen administered to prepartum cows, and found that calves born to Ribotan-treated dams showed the greatest increases in daily weight gain (up to 35.8% above control) and all-group 100% livability, outperforming calves from groups receiving the other immunostimulants. Similarly, Krytsia et al. [50], working with sport horse foals, reported that Ribotan produced more pronounced improvements in cellular immunity parameters than other immunomodulators, attributing this to its dual action on both T-cell and B-cell immunity. Sodium nucleinate, primarily acting through stimulation of leukocyte reactions and nucleic-acid metabolism, produced qualitatively similar but quantitatively more moderate improvements. Dinardo et al. [18] likewise reported that direct nucleotide supplementation in calves stimulated immune responses but yielded a more modest magnitude of effect compared to polypeptide-based preparations, consistent with its predominantly leukocyte-mediated mechanism of action as characterized for sodium nucleinate [51]. Both preparations, however, clearly outperformed the control, supporting the rationale for targeted prepartum immunomodulation of cows as a tool to improve neonatal calf quality in commercial dairy herds.

Several limitations must be acknowledged. The study was performed on a single commercial farm under the specific zoohygienic and seasonal microclimate conditions of West Kazakhstan, which—as our seasonal data show—influence humidity, CO_2_ accumulation, and microbial load, and may therefore modulate the magnitude of the observed effects. Specific IgG concentrations, colostrum quality, and pathogen-specific antibody titers were not measured directly; their inference from total protein and γ-globulin dynamics, although biologically well supported, should be confirmed in future work using ELISA-based immunoglobulin quantification. Long-term effects on reproductive performance and first-lactation milk yield, which are strongly linked to early-life health, also remain to be evaluated.

Despite these limitations, our findings clearly demonstrate that a single prepartum injection of Ribotan or sodium nucleinate to Holstein cows significantly improves early-life adaptation, growth, immune competence, and metabolic status of calves, with Ribotan providing the most pronounced benefit. This supports the practical use of such immunomodulators as a complementary tool alongside high-quality colostrum management and optimal zoohygienic conditions in modern dairy production systems.

## 5. Conclusions

Prepartum administration of immunotropic preparations to Holstein cows positively influenced the physiological and metabolic status of their offspring during the first 60 days of life. Calves born to treated cows exhibited significantly greater body weight and average daily gain compared with the control group, indicating improved growth performance. The treatments also enhanced protein metabolism and immune-related parameters, as evidenced by increased concentrations of total protein, albumin, and γ-globulins, together with higher leukocyte counts. In addition, treated calves showed lower cortisol concentrations during the postnatal period, suggesting improved adaptation and reduced physiological stress.

Among the tested preparations, Ribotan generally produced the most pronounced effects, while sodium nucleinate also resulted in consistent improvements compared with the control group.

PCA and correlation analysis further demonstrated that growth, metabolic activity, and immune function are tightly interconnected processes. Variables associated with anabolic metabolism and productivity (body mass, albumin, total protein, γ-globulins) were positively associated and contributed to the separation of treated groups, whereas stress-related indicators (cortisol and α-globulins) were inversely related to growth performance.

Overall, Ribotan exhibited the most pronounced biological effect, while sodium nucleinate showed a moderate but consistent positive influence. The obtained results indicate that the prepartum immunomodulation of cows is an effective strategy to enhance the early-life adaptation, metabolic efficiency, and growth of calves. These findings may have practical significance for improving calf rearing efficiency and reducing early-life morbidity under commercial dairy farm conditions.

## Figures and Tables

**Table 1:** Feeding regimen and daily ration composition.

Feed Component	Lactating Cows (kg/Day)	Dry Cows (kg/Day)	Pregnant Heifers (kg/Day)	Heifer Calves Up to 6 Months (kg/Day)	Bull Calves Up to 6 Months (kg/Day)	Heifers Older Than 6 Months (kg/Day)	Bulls Older Than 6 Months (kg/Day)
Silage	23	18	18	–	–	12	12
Alfalfa hay	4	–	–	3	3	2	2
Straw	–	3	2.5	–	–	–	–
Barley grain	5	2.5	2.5	1.5	1.5	2.5	5
Maize grain	2.5	–	–	–	–	–	–
Rapeseed meal	2.5	–	–	–	–	–	–
Premix	0.15	0.05	–	0.05	–	–	–
Tricalcium phosphate	0.2	–	–	–	–	–	–
Whole milk	–	–	–	5	5	–	–

Data are expressed as kilograms per animal per day (kg/day). The en dash (–) indicates that the corresponding feed component was not included in the ration.

**Table 2:** Growth performance of calves.

Days	1			10			30			60			*p*-Value		
Parameters	Control	Ribotan	Sodium Nucleinate	Control	Ribotan	Sodium Nucleinate	Control	Ribotan	Sodium Nucleinate	Control	Ribotan	Sodium Nucleinate	By Group	By Day	By Group × Day
Body mass, kg	33.7 ± 0.17 a	33.8 ± 0.17 a	33.9 ± 0.17 a	39.23 ± 0.21 b	40.03 ± 0.21 a	39.8 ± 0.26 ab	51.47 ± 0.32 b	52.87 ± 0.32 a	52.73 ± 0.38 a	72.4 ± 0.36 b	74.83 ± 0.50 a	74.5 ± 0.46 a	NS	0.0001	0.0001
AVG, g	-	-	-	-	-	-	592 ± 10.82 b	636 ± 15.10 a	628 ± 17.09 a	698 ± 12.12 b	732 ± 11.53 a	725 ± 14.53 a	NS	0.0001	NS

Data are presented as mean ± SEM. Different superscript letters (a,b) within the same row and sampling day indicate significant differences among treatment groups according to Tukey’s HSD test (*p* ≤ 0.05). *p*-values are presented for the effects of treatment group, sampling day, and treatment × day interaction. NS—non-significant (*p* ≥ 0.05).

**Table 3:** Hematological parameters of calves.

Days	1			10			30			60			*p*-Value		
Parameters	Control	Ribotan	Sodium Nucleinate	Control	Ribotan	Sodium Nucleinate	Control	Ribotan	Sodium Nucleinate	Control	Ribotan	Sodium Nucleinate	By Group	By Day	By Group × Day
RBC	7.03 ± 0.21 a	6.60 ± 0.20 ab	6.43 ± 0.40 b	6.30 ± 0.30 a	5.63 ± 0.15 b	6.03 ± 0.25 ab	6.47 ± 0.35 a	5.57 ± 0.35 b	6.30 ± 0.30 a	6.77 ± 0.25 a	6.50 ± 0.30 a	6.67 ± 0.25 a	0.0389	0.0001	NS
Hemoglobin, g/L	87.33 ± 2.52 a	87.00 ± 1.00 a	86.67 ± 6.51 a	88.67 ± 2.52 a	86.00 ± 1.00 ab	84.33 ± 2.52 b	77.33 ± 2.52 ab	73.67 ± 1.53 b	79.00 ± 2.00 a	77.33 ± 2.52 a	78.00 ± 2.00 a	79.33 ± 2.52 a	NS	0.0167	NS
WBC	7.73 ± 0.50 c	12.00 ± 0.20 a	9.70 ± 0.40 b	9.23 ± 0.45 b	12.17 ± 0.15 a	11.03 ± 0.55 ab	9.03 ± 0.45 b	9.93 ± 0.35 ab	10.20 ± 0.40 a	8.70 ± 0.30 b	9.50 ± 0.30 a	9.10 ± 0.30 ab	0.0021	0.0022	0.0001

Data are presented as mean ± SEM. Different superscript letters (a,b) within the same row and sampling day indicate significant differences among treatment groups according to Tukey’s HSD test (*p* ≤ 0.05). *p*-values are presented for the effects of treatment group, sampling day, and treatment × day interaction. NS—non-significant (*p* ≥ 0.05).

**Table 4:** Biochemical parameters of calves.

Days	1			10			30			60			*p*-Value		
Parameters	Control	Ribotan	Sodium Nucleinate	Control	Ribotan	Sodium Nucleinate	Control	Ribotan	Sodium Nucleinate	Control	Ribotan	Sodium Nucleinate	By Group	By Day	By Group × Day
Total protein, g/L	61.97 ± 0.55 b	73.40 ± 0.40 a	73.60 ± 2.10 a	60.80 ± 1.00 b	68.40 ± 1.90 a	67.60 ± 1.60 a	59.77 ± 1.25 b	64.10 ± 1.20 a	61.50 ± 1.80 ab	60.97 ± 1.15 b	65.20 ± 1.20 a	62.50 ± 1.80 ab	0.0006	0.0001	0.0001
Albumin, g/L	21.40 ± 0.40 b	25.10 ± 0.30 a	24.90 ± 1.10 a	22.60 ± 0.50 b	24.80 ± 0.50 a	23.80 ± 0.70 ab	27.90 ± 0.50 b	30.60 ± 0.80 a	29.50 ± 0.40 ab	28.00 ± 0.30 b	30.70 ± 0.80 a	29.90 ± 0.30 ab	0.0170	0.0001	NS
α-globulins, %	18.77 ± 0.95 a	18.70 ± 0.40 a	17.40 ± 1.00 a	17.20 ± 1.30 a	15.60 ± 0.60 ab	15.10 ± 0.10 b	14.50 ± 0.50 a	12.57 ± 0.55 ab	12.10 ± 1.10 b	10.10 ± 0.10 a	7.90 ± 0.30 b	8.10 ± 0.10 b	NS	0.0001	NS
β-globulins, %	5.80 ± 0.40 b	6.97 ± 0.15 a	6.10 ± 0.50 ab	7.60 ± 0.90 b	8.73 ± 0.45 ab	9.30 ± 0.60 a	7.07 ± 0.35 b	8.90 ± 0.60 a	8.00 ± 0.80 ab	6.90 ± 0.20 a	6.80 ± 0.40 ab	6.60 ± 0.60 b	0.0315	0.0001	0.0187
γ-globulins, %	16.00 ± 1.00 b	25.40 ± 0.50 a	25.20 ± 2.40 a	13.37 ± 0.55 b	19.27 ± 0.55 a	19.33 ± 1.55 a	9.97 ± 0.45 b	12.00 ± 0.60 a	11.83 ± 0.85 a	16.00 ± 0.40 b	19.80 ± 0.40 a	17.90 ± 1.40 ab	0.0022	0.0001	0.0001
Glucose, mmol/L	4.30 ± 0.20 a	4.60 ± 0.20 a	4.50 ± 0.20 a	4.60 ± 0.20 a	4.80 ± 0.20 a	4.73 ± 0.25 a	3.80 ± 0.10 b	4.40 ± 0.20 a	4.20 ± 0.20 a	3.00 ± 0.20 c	3.90 ± 0.10 a	3.70 ± 0.10 b	0.0044	0.0001	NS
Urea, mmol/L	3.40 ± 0.10 a	3.50 ± 0.10 a	3.50 ± 0.10 a	3.50 ± 0.10 b	3.73 ± 0.15 a	3.70 ± 0.10 ab	3.40 ± 0.20 a	3.20 ± 0.10 b	3.30 ± 0.10 b	3.60 ± 0.20 a	3.27 ± 0.15 b	3.30 ± 0.10 b	NS	0.0001	0.0123
Calcium, mmol/L	2.90 ± 0.20 a	3.00 ± 0.10 a	2.97 ± 0.15 a	2.87 ± 0.15 b	3.00 ± 0.10 a	2.90 ± 0.10 ab	2.73 ± 0.15 b	2.90 ± 0.10 a	2.80 ± 0.10 ab	2.40 ± 0.10 b	3.10 ± 0.10 a	2.97 ± 0.15 ab	0.0001	NS	0.0040
Phosphorus, mmol/L	1.70 ± 0.10 a	1.87 ± 0.06 a	1.80 ± 0.10 a	1.70 ± 0.10 a	1.80 ± 0.10 a	1.80 ± 0.10 a	1.63 ± 0.06 a	1.77 ± 0.06 a	1.73 ± 0.06 a	1.60 ± 0.10 b	1.97 ± 0.15 a	1.93 ± 0.15 a	0.0001	NS	NS
Cortisol, nmol/L	150 ± 2 c	178 ± 2 a	166 ± 2 b	53 ± 3 c	65 ± 2 a	58 ± 2 b	40.67 ± 2.52 b	46 ± 2 a	44.33 ± 1.53 ab	33 ± 2 b	37.67 ± 1.53 a	37 ± 2 a	NS	0.0001	0.0001

Data are presented as mean ± SEM. Different superscript letters (a–c) within the same row and sampling day indicate significant differences among treatment groups according to Tukey’s HSD test (*p* ≤ 0.05). *p*-values are presented for the effects of treatment group, sampling day, and treatment × day interaction. NS—non-significant (*p* ≥ 0.05).

## Data Availability

The original contributions presented in this study are included in the article. Further inquiries can be directed to the corresponding author.

## Abbreviations

The following abbreviations are used in this manuscript:

ADG	Average daily gain
RBC	Red blood cells
WBC	White blood cells
PCA	Principal component analysis
IgG	Immunoglobulin G
ANOVA	Analysis of variance

## References

[1] Abugaliyev S.K., Yuldashbayev Y.A., Baimukanov A.D., Bupebayeva L.R. (2019). Efficient methods in breeding dairy cattle of the Republic of Kazakhstan. Sci. J. Pedag. Econ..

[2] Bekenov D.M., Spanov A.A., Sultanbai D.T., Zhaksylykova G.K., Baimukanov A.D. (2019). The effect of canola meal application in the diet of dairy cows of Holstein breed in «Bayserke Agro» LLP. Acad. J. Phys. Chem. Sci..

[3] Yelemesov K.Y., Baimukanov A.D. (2020). The estimated breeding value of servicing bulls of domestic breeds by offspring quality using the BLUP method. Sci. J. Pedag. Econ..

[4] Kazhgaliyev N.Z., Titanov Z., Ateikhan B., Sharapatov T.S., Gabbassov M.B., Seiteuov T.K., Burambayeva N.B., Temirzhanova A.A. (2023). Maternal instinct of imported meat-direction cattle and ethology of their calves. J. Anim. Behav. Biometeorol..

[5] Kazhgaliyev N., Nurgulsim K., Gabbassov M., Makhanbetova A., Zhanabayev A., Terlikbayev A., Assanbayev T., Toishimanov M., Sharapatov T. (2026). Gene-polymorphism effects on growth efficiency in the Kalmyk breed of Central Asia. Genes.

[6] Mee J.F. (2023). Invited review: Bovine neonatal morbidity and mortality—Causes, risk factors, incidences, sequelae and prevention. Reprod. Domest. Anim..

[7] Sivula N.J., Ames T.R., Marsh W.E., Werdin R.E. (1996). Descriptive epidemiology of morbidity and mortality in Minnesota dairy heifer calves. Prev. Vet. Med..

[8] Besser T.E., Gay C.C. (1999). Failure of passive transfer in calves. Am. Assoc. Bov. Pr. Conf. Proc..

[9] Michigan State University Extension (2023). Transfer of Passive Immunity in Calves: Ensuring Calf Health and Profitability. https://www.canr.msu.edu/news/transfer-of-passive-immunity-in-calves-ensuring-calf-health-and-profitability.

[10] Weaver D.M., Tyler J.W., VanMetre D.C., Hostetler D.E., Barrington G.M. (2000). Passive transfer of colostral immunoglobulins in calves. J. Vet. Intern. Med..

[11] Morrill K.M., Marston S.P., Whitehouse N.L., Van Amburgh M.E., Schwab C.G., Haines D.M., Erickson P.S. (2010). Anionic salts in the prepartum diet and addition of sodium bicarbonate to colostrum replacer, and their effects on immunoglobulin G absorption in the neonate. J. Dairy Sci..

[12] Skibiel A.L., Fabris T.F., Corrá F.N., Torres Y.M., McLean D.J., Chapman J.D., Kirk D.J., Dahl G.E., Laporta J. (2017). Effects of feeding an immunomodulatory supplement to heat-stressed or actively cooled cows during late gestation on postnatal immunity, health, and growth of calves. J. Dairy Sci..

[13] Anton M.V., Bernhardt H., Steinhoff-Wagner J. (2026). Growth during the first week of life and physiological body weight oscillation between feedings using high-frequency weighing in individually housed calves fed unrestricted amounts of milk twice daily. J. Dairy Sci..

[14] Cid de la Paz M., Viquez-Umana F., Mancheno M., Fernandez-Wallace T., Mantovani H.C., Cangiano L.R. (2025). Exploring the impacts of colostrum on systemic immune development in dairy calves. J. Dairy Sci..

[15] Bierlein G.F., Gross J.J. (2025). Physiological changes during the evolution of diarrhea in preweaning calves prior to the onset of clinical signs. J. Dairy Sci..

[16] Urie N.J., Lombard J.E., Shivley C.B., Kopral C.A., Adams A.E., Earleywine T.J., Olson J.D., Garry F.B. (2018). Preweaned heifer management on US dairy operations: Part V. Factors associated with morbidity and mortality in preweaned dairy heifer calves. J. Dairy Sci..

[17] Foster D.M., Smith G.W. (2009). Pathophysiology of diarrhea in calves. Vet. Clin. North Am. Food Anim. Pract..

[18] Sato H., Koiwa M. (2008). Fecal D- and L-lactate, succinate and volatile fatty acid levels, and relationships with fecal acidity and diarrhea in neonatal calves. Anim. Sci. J..

[19] Dudek K., Bednarek D., Ayling R.D., Szacawa E. (2014). Stimulation and analysis of the immune response in calves from vaccinated pregnant cows. Res. Vet. Sci..

[20] Bertagnon H.G., Depaoli C.R., Oliveira S.N., Milla B., Zdepski B.F., Garbossa G. (2024). Immunostimulation of bronchoalveolar response in calves vaccinated against bovine respiratory disease. Pesqui. Vet. Bras..

[21] Dinardo F.R., Maggiolino A., Martinello T., Liuzzi G.M., Elia G., Zizzo N., Latronico T., Mastrangelo F., Dahl G.E., De Palo P. (2022). Oral administration of nucleotides in calves: Effects on oxidative status, immune response, and intestinal mucosa development. J. Dairy Sci..

[22] Smirnov Y.P., Suvorova I.L. (2017). Prevention Method of Postnatal Infection by Bovine Leukosis Virus of Young Bovine Stock (Patent RU2621146C1). https://patents.google.com/patent/RU2621146C1/en.

[23] Smolentsev S., Bogomolova O., Fedorov Y., Markova E., Neminuschaya L., Skotnikova T., Eremets V., Melnik R., Melnik N., Lyulkova L., Zokirjon ugli K.S., Muratov A., Ignateva S. (2023). Evaluation of new biological products in calf rearing. Fundamental and Applied Scientific Research in the Development of Agriculture in the Far East (AFE-2022).

[24] Baimukanov A.D., Bissembayev A.T., Yuldashbayev Y.A., Chindaliyev A.E., Shamshidin A.S., Amerkhanov K.A., Saginbayev A.K., Aubakirov K.A. (2024). Reproductive indicators of the Alatau cattle breed of Kazakhstan population. Online J. Biol. Sci..

[25] Immler M., Büttner K., Gärtner T., Wehrend A., Donat K. (2022). Maternal Impact on Serum Immunoglobulin and Total Protein Concentration in Dairy Calves. Animals.

[26] Roland L., Drillich M., Klein-Jöbstl D., Iwersen M. (2016). Invited review: Influence of climatic conditions on the development, performance, and health of calves. J. Dairy Sci..

[27] Tao S., Dahl G.E. (2013). Invited review: Heat stress effects during late gestation on dry cows and their calves. J. Dairy Sci..

[28] Murayama K., Kobayashi N., Nishizawa N., Oba M., Sugino T. (2024). Evaluation of serum concentrations of total protein and gamma-globulin as an indicator of serum immunoglobulin G concentration in dairy calves. JDS Commun..

[29] United States Department of Agriculture Animal and Plant Health Inspection Service (2021). Colostrum Feeding and Passive Immunity in U.S. Dairy Heifer Calves (Info Brief, August 2021). https://www.aphis.usda.gov/sites/default/files/colostrum-feeding-passive-immunity-heifer-calves.pdf.

[30] Godden S. (2008). Colostrum management for dairy calves. Vet. Clin. N. Am. Food Anim. Pract..

[31] Chantillon L., Pas M.L., Vlaminck L., Pardon B. (2024). Long-Term Survival in 241 Cases of Intussusception in Cattle and Factors Associated with Mortality. Animals.

[32] Guo Y.-Q., Hu Y.-R., Liu S.-R., Wang M., Xian Z.-Y., Liu D.-W., Sun B.-L., Li Y.-K., Liu G.-B., Deng M. (2023). Effects of the Oat Hay Feeding Method and Compound Probiotic Supplementation on the Growth, Antioxidant Capacity, Immunity, and Rumen Bacteria Community of Dairy Calves. Antioxidants.

[33] Mohamadi Roodposhti P., Dabiri N. (2012). Effects of probiotic and prebiotic on average daily gain, fecal shedding of *Escherichia coli*, and immune system status in newborn female calves. Asian-Australas. J. Anim. Sci..

[34] Brasil M.J., Cardin J.L., Vagnoni D.B., Macias-Rioseco M., Kim S., Van Kessel J.S., Haley B.J., Nichols C.A., Rossow H.A. (2026). Effects of probiotic supplementation on preweaning Holstein × Angus calf performance and health. Appl. Anim. Sci..

[35] Cortese V.S., Kirkpatrick M.A., Short T.H., Voortman B. (2020). Effect of Serum Total Protein Concentration on Early-Life Health and Growth of Dairy Calves. J. Am. Vet. Med. Assoc..

[36] Aghakhani M., Shahraki A.D.F., Tabatabaei S.N., Toghyani M., Moosavi-Zadeh E., Rafiee H. (2023). 24-Hour Postnatal Total Serum Protein Concentration Affects the Health and Growth Performance of Female Holstein Dairy Calves. Vet. Med. Sci..

[37] Rainard P., Gilbert F.B., Germon P. (2022). Immune defenses of the mammary gland epithelium of dairy ruminants. Front. Immunol..

[38] Dudek K., Szacawa E., Bednarek D. (2024). The effect of pegbovigrastim administration on the nonspecific immunity of calves. J. Vet. Intern. Med..

[39] Egli C.P., Blum J.W. (1998). Clinical, haematological, metabolic and endocrine traits during the first three months of life of suckling Simmentaler calves held in a cow–calf operation. J. Vet. Med. A.

[40] Brun-Hansen H.C., Kampen A.H., Lund A. (2006). Hematologic values in calves during the first 6 months of life. Vet. Clin. Pathol..

[41] Mohri M., Sharifi K., Eidi S. (2007). Hematology and serum biochemistry of Holstein dairy calves: Age-related changes and comparison with blood composition in adults. Res. Vet. Sci..

[42] Nedjma A., Metref A.K., Saidan K. (2025). Changes in values of serum protein electrophoresis by body condition score, physiological stage and age in healthy cattle. Large Anim. Rev..

[43] Munang’andu H.M., Mudronova D., Popelka P. (2024). Editorial: Natural Immunomodulators in Veterinary Medicine. Front. Vet. Sci..

[44] Ockenden E.M., Russo V.M., Leury B.J., Giri K., Wales W.J. (2023). Preweaning Nutrition and Its Effects on the Growth, Immune Competence and Metabolic Characteristics of the Dairy Calf. Animals.

[45] Urbutis M., Malašauskienė D., Televičius M., Juozaitienė V., Baumgartner W., Antanaitis R. (2023). Evaluation of the metabolic relationship between cows and calves by monitoring calf health and cow automatic milking system and metabolic parameters. Animals.

[46] Zhang N., Teng Z., Li P., Fu T., Lian H., Wang L., Gao T. (2021). Oscillating dietary crude protein concentrations increase N retention of calves by affecting urea-N recycling and nitrogen metabolism of rumen bacteria and epithelium. PLoS ONE.

[47] Kirovski D. (2015). Endocrine and Metabolic Adaptations of Calves to Extra-Uterine Life. Acta Vet.-Beogr..

[48] Arfuso F., Minuti A., Liotta L., Giannetto C., Trevisi E., Piccione G., Lopreiato V. (2023). Stress and inflammatory response of cows and their calves during peripartum and early neonatal period. Theriogenology.

[49] Masmeijer C., Deprez P., van Leenen K., De Cremer L., Cox E., Devriendt B., Pardon B. (2021). Arrival Cortisol Measurement in Veal Calves and Its Association with Body Weight, Protein Fractions, Animal Health and Performance. Prev. Vet. Med..

[50] Krytsia I. (2016). The Influence of Immunomodulators on the Performance of Cellular Immunity at the Foals of Saddle Breeds. Sci. Messenger LNU Vet. Med. Biotechnol..

[51] Zemskov A.M., Sitnikova V.P., Trutnev B.D., Morozova V.P., Kryukov V.M., Nikitin A.V., Yevstratova E.F., Nastausheva T.L. (1990). The effect of sodium nucleinate on allergic and immunological reactions. J. Hyg. Epidemiol. Microbiol. Immunol..

